# Creatine monohydrate supplementation on lower-limb muscle power in Brazilian elite soccer players

**DOI:** 10.1186/1550-2783-11-32

**Published:** 2014-06-18

**Authors:** João G Claudino, Bruno Mezêncio, Sérgio Amaral, Vinícius Zanetti, Fabiana Benatti, Hamilton Roschel, Bruno Gualano, Alberto C Amadio, Julio C Serrão

**Affiliations:** 1 School of Physical Education and Sport - Laboratory of Biomechanics, University of Sao Paulo, Av Mello de Moraes, 65, Sao Paulo, SP 05508-030, Brazil; 2 Department of Health and Performance, Red Bull Brazil Football, João Lúcio do Prado street, Km 10, Jarinu, SP 13240-000, Brazil; 3 School of Physical Education and Sport - Laboratory of Applied Nutrition and Metabolism, University of Sao Paulo, Av Mello de Moraes, 65, Sao Paulo, SP 05508-030, Brazil; 4 School of Physical Education and Sport - Laboratory of Neuromuscular Adaptations to Strength Training, University of Sao Paulo, Av Mello de Moraes, 65, Sao Paulo, SP 05508-030, Brazil; 5 School of Medicine - Division of Rheumatology, University of Sao Paulo, Av Mello de Moraes, 65, Sao Paulo, SP 05508-030, Brazil

**Keywords:** Football, Team sports, Dietary supplement, Jumping, Athletes

## Abstract

**Background:**

Studies involving chronic creatine supplementation in elite soccer players are scarce. Therefore, the aim of this study was to examine the effects of creatine monohydrate supplementation on lower-limb muscle power in Brazilian elite soccer players (n = 14 males) during pre-season training.

**Findings:**

This was a randomized, double-blind, placebo-controlled parallel-group study. Brazilian professional elite soccer players participated in this study. During the pre-season (7 weeks), all the subjects underwent a standardized physical and specific soccer training. Prior to and after either creatine monohydrate or placebo supplementation, the lower-limb muscle power was measured by countermovement jump performance. The Jumping performance was compared between groups at baseline (p = 0.99). After the intervention, jumping performance was lower in the placebo group (percent change = - 0.7%; ES = - 0.3) than in the creatine group (percent change = + 2.4%; ES = + 0.1), but it did not reach statistical significance (p = 0.23 for time x group interaction). Fisher’s exact test revealed that the proportion of subjects that experienced a reduction in jumping performance was significantly greater in the placebo group than in the creatine group (5 and 1, respectively; p = 0.05) after the training. The magnitude-based inferences demonstrated that placebo resulted in a *possible negative effect* (50%) in jumping performance, whereas creatine supplementation led to a *very likely trivial effect* (96%) in jumping performance in the creatine group.

**Conclusions:**

Creatine monohydrate supplementation prevented the decrement in lower-limb muscle power in elite soccer players during a pre-season progressive training.

## Background

The creatine/phosphorylcreatine system can provide energy when the rate of ATP utilization outstrips the rate of production by mitochondrial respiration, maintaining ATP homeostasis at specific sites of high energy turnover. Additionally, it may function as an ATP “shuttle”, transferring mitochondrial ATP to the cytosol [1]. Increased levels of creatine/phosphorylcreatine via creatine supplementation have been consistently shown to increase performance in high-intensity intermittent exercise [2-6]. Not surprisingly, creatine supplementation has been largely used by athletes engaged in multiple-sprint events, such as soccer [7] and other team sports [8].

In fact, it has been shown that the ability to accelerate, perform maximal intermittent sprints, and to jump are required for the high-level soccer performance [9]. Therefore, creatine supplementation has been considered as a potential ergogenic strategy to improve muscle power capacity in this sport. However, despite the great popularity of creatine supplements among high-level athletes, chronic studies (i.e., > 7 days) involving soccer players remain scarce. Creatine supplementation for 7 days improved performance in a soccer-specific battery of tests, including a dribble test, a sprint-power test, an endurance test, and a vertical jump test [10]. Supporting these findings, it was shown that 6 days of creatine supplementation improved repeated sprint performance and jumping ability after an intermittent exercise test in highly trained soccer players [11]. Furthermore, beneficial effects of 6 days of creatine supplementation were observed on repeated sprint and agility tasks in elite female soccer players [12]. To the best of our knowledge, only 1 study investigated the chronic effects of creatine supplementation along with training in soccer players [13]. These authors showed that 13 weeks of creatine supplementation (2 × 7.5 g/d in the first week and 5 g/d throughout the rest of the protocol) improved muscle strength but not lean mass in collegiate female soccer players [13].

Therefore, the aim of this study was to examine the effects of creatine supplementation on lower-limb muscle power in Brazilian elite soccer players during their initial phase of the pre-season training period. Given that during this period, the training loads are intensified, usually leading to a functional overreaching (i.e., a small decrement in performance) [14]. We expected that creatine supplementation would improve or, at least, mitigate the decline in lower-limb muscle power performance.

## Methods

### Experimental design

This was a randomized, double-blind, placebo-controlled parallel-group study. Brazilian elite soccer players participated in this study. In order to evaluate lower-limb muscle power, countermovement jump (CMJ) performance was assessed using a strain-gauge force plate. During the initial phase of the pre-season (7 weeks), all of the subjects underwent a standardized physical and specific training previously determined by the team’s trainers. Prior to and after either creatine or placebo supplementation, CMJ, dietary intake, and anthropometric parameters (i.e., body mass and height) were assessed.

### Subjects

Twenty three Brazilian elite soccer players from the same soccer team (Red Bull Brazil Football, Sao Paulo, Brazil) participated in this study. Five subjects were discharged from the team during the study, 3 had injuries, and 1 refused to supplement. Hence, 14 (player positions = 5 defenders, 3 midfielders, and 6 forwards) male subjects (18.3 ± 0.9 years; 69.9 ± 8.8 kg; 1.75 ± 0.1 m) completed the trial and were analyzed. Thus, 7 subjects remained in the Placebo Group and 7 in the Creatine Group. None of them declared using dietary supplements for at least 3 months before the baseline. All of the subjects underwent the same diet and training schedules during the protocol. The experimental procedures were approved by the University of Sao Paulo Institutional Review Board for Human Subjects, and a written informed consent was obtained prior to their participation.

### Training protocol

The protocol during the pre-season was comprised of both resistance training and specific training. Resistance training was a hypertrophy-oriented training supervised by a strength and conditioning coach, following classical recommendations [15]. Resistance exercise sessions were performed twice a week and lasted between 50 and 60 minutes, and involved multiple joint exercises (i.e., squat, bench press, lat pull down, leg press, and seated shoulder press) with 3 × 8–10 repetition maximum interspersed by 1 to 3 minutes of recovery. Additionally, plyometric exercises were performed (i.e., horizontal, vertical, and depth jumping) during resistance training sessions, as this type of training can positively affect lower-limb power [16]. The specific training consisted of small-sided games (*e.g.,* passing, shooting, offense and defense drills as well as game simulations) performed 4 to 5 times a week. Training regimen involved 6 ± 1 sessions per week, with each session lasting 1.4 ± 0.2 hours.

The training load was determined for each training mode (i.e.; resistance training and specific training). The resistance training load was determined according to previous criteria by multiplying the RPE score which was reported 30 minutes after the end of the training session using the modified 10-point Borg scale - CR-10: RPE (session RPE) by the training volume (i.e., number of sets X number of repetitions) [17]. The training load of the specific training was also assessed according to previous criteria by multiplying the session RPE by the training volume (i.e.; duration, in minutes, of the training session) [18]. Total training load, hereafter called training load, was measured as the summation (in arbitrary units) of the specific training loads and the resistance training loads per week according to previously described criteria [19].

**Figure 1:**
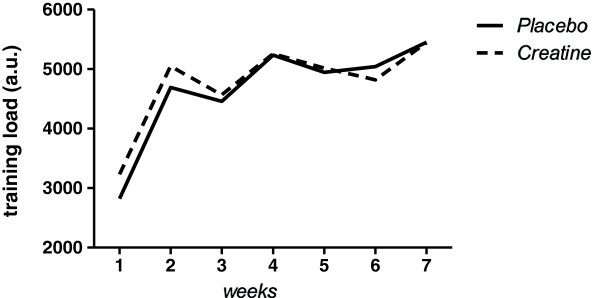
**Illustration of the training load (as determined by the RPE method **[19]**) progression throughout the intervention period.**

Training load, as determined by RPE method [19], was progressively increased throughout the training period as depicted in Figure 1.

### Jumping test

CMJ performance assessment protocol consisted of 8 jumps with 60-second intervals between each attempt [20,21]. The average of the 8 jumps was considered for analysis. CMJ was initiated from a standing position. Subjects were instructed to maintain their hands on their chest and freely determine the amplitude of the countermovement in order to avoid changes in jumping coordination [22]. Subjects were encouraged to jump as high as possible. Previous reports support the use of jumping to measure the effects of creatine on lower limb performance [10,23-25].

A strain-gauge force plate (AMTI BP600900; Watertown, EUA) was used to measure jumping performance. Data referring to the vertical ground reaction force component (Fy) were collected at a 1000 Hz. A Butterworth low pass (90 Hz cut off frequency) on-line filtering was also performed. Jumping height was determined by the impulse. The jumping performance was calculated by the following equation:

$$h=v^{2}/2g$$

where *h* is the height of jump, *v* is the vertical takeoff velocity, and *g* is the acceleration due to gravity. The data were analysed through the MatLab R2009b software (Mathworks, EUA).

### Dietary intake

Dietary intake was assessed by means of 3, 24-hour dietary recalls undertaken on separate days (2 week days and 1 weekend day) using a visual aid photo album of real foods. Energy, macronutrient and creatine intake were analyzed by the software Virtual Nutri (Sao Paulo, Brazil). Supplementary creatine was not considered in the analysis.

### Creatine supplementation protocol and blinding procedure

The subjects from the creatine group received 20 g/d of creatine monohydrate (Probiótica, Sao Paulo, Brazil) for 1 week divided into 4 equal doses, followed by single daily doses of 5 g for the next 6 weeks. The subjects from the placebo group were given the same dose of dextrose. During the loading phase, supplements were presented in 4 packages and subjects were instructed to ingest the packet contents at breakfast, lunch, dinner and before bedtime. During the maintenance phase, the subjects consumed the supplement as a single dose during their lunch. They were asked to dissolve the supplements preferably in juice, in order to mask the supplements. The compliance to creatine supplementation was monitored weekly by personal communication, as previously done in our studies in which creatine supplementation was shown to be capable of increasing muscle phosphorylcreatine content [26-28]. The supplement packages were coded, so that, neither the investigators nor the participants were aware of the contents until completion of the analyses. The supplements were provided by a staff member of our research team who did not have any participation in the data acquisition, analyses, and interpretation. In order to verify the purity of the creatine monohydrate used, a sample was analyzed by HPLC and purity was established as 99.9%.

### Anthropometric measurements

At baseline and after the intervention, body mass and height were measured using standardized procedures, with a calibrated scale (i.e., ± 0.1 Kg) and a stadiometer (Filizola, Brasil).

### Statistical analysis

Data were tested for normality and sphericity by Kolmogorov-Smirnov and Mauchly tests, respectively. A mixed model test was used to assess possible changes in the dependent variables. A Tukey post-hoc was used if necessary. Fisher’s exact test was used to compare the possible differences between groups in the proportion of subjects who correctly guessed their supplements as well as in the incidence of performance reduction. Cohen’s effect sizes (ES) were calculated for each group. The significance level was previously set at p < 0.05. In addition, jumping performance data were analyzed using a contemporary magnitude-based inferences approach [29] in order to detect small effects of practical importance in an applied setting, a technique which is becoming increasingly common in an exercise performance research [30-33]. This uses a spread sheet to establish the likelihood (percentually) of each experimental manipulation having a positive/trivial/negative effect. A Cohen’s unit of 0.2 was employed as the smallest meaningful change in performance. Where the chance of benefit or harm were both >5%, the true effect was deemed *unclear*. Qualitative descriptors were assigned to the quantitative percentile scores as follows: 25-75% *possible*; 75-95% *likely*; 95-99% *very likely*; >99% *almost certain*[34,35]. Data are expressed as mean ± SD, unless otherwise stated.

## Results

Anthropometric characteristics were not significantly different between groups at baseline (p > 0.05). Body mass was comparable between the creatine and the placebo groups. After the intervention, both groups tended to increase body mass (creatine: percent change = + 0.8; ES = + 0.1 and placebo: percent change = + 2.2%; ES = + 0.1, main time effect p = 0.06), with no significant differences between them (group × time interaction p = 0.7).

At the end of the study, subjects were inquired about the substance ingested. The percentage of correct answers was compared between groups as a way of ensuring the efficiency of blinding. Four subjects correctly identified the supplement in the creatine group, whereas 2 subjects were able to identify the correct supplement in the placebo group (p = 0.29). Dietary intake (Table 1) did not differ significantly within- or between-groups.

**Table 1:** Dietary intake in soccer players supplemented with either creatine or placebo during pre-season training

	**Placebo (n = 7)**		**Creatine (n = 7)**	
	**Pre**	**Post**	**Pre**	**Post**
Total Energy (Kcal/d)	2887.9 ± 700.6	2952.2 ± 634.4	2718.4 ± 603.2	3035.1 ± 943.2
Carbohydrate (g/d)	379.2 ± 108.9	451.1 ± 143.9	361.8 ± 90.4	462.0 ± 147.6
Lipids (g/d)	98.0 ± 26.7	79.5 ± 16.2	92.1 ± 23.6	81.9 ± 33.7
Protein (g/d)	122.3 ± 28.9	108.2 ± 23.8	110.5 ± 12.7	112.4 ± 42.1
Protein (g/Kg body mass/d)	1.8 ± 0.5	1.6 ± 0.4	1.6 ± 0.2	1.7 ± 0.7
Creatine (g/d)	1.2 ± 0.4	1.2 ± 0.4	1.5 ± 0.7	1.2 ± 0.4

There were no significant differences within- or between-groups.

**Figure 2:**
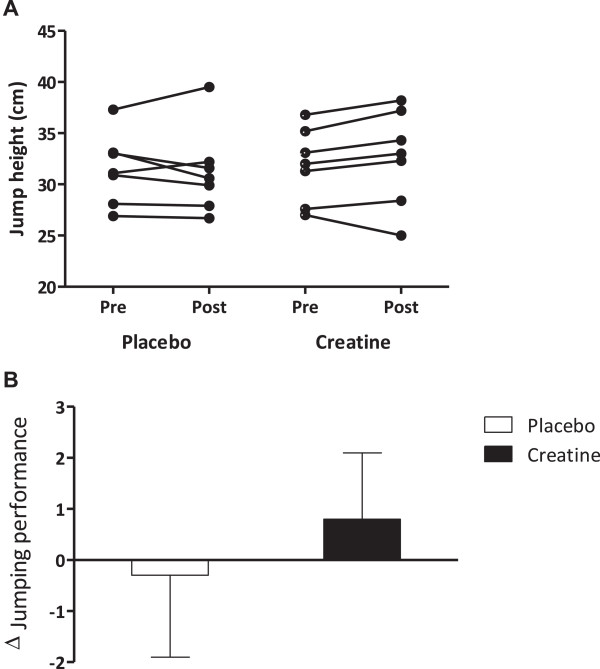
**Jumping performance before (Pre) and after 7 weeks (Post) of either creatine (n = 7) or placebo (n = 7) supplementation in soccer players during pre-season training. Panel A**: individual data. **Panel B**: mean ± standard deviation of delta. No significant difference between groups across time (group x time interaction) was observed (p = 0.23).

Jumping performance (Figure 2) was comparable between groups at baseline (p = 0.99). After the intervention, jumping performance was lower in the placebo group (percent change = - 0.7%; ES = - 0.3) than in the creatine group (percent change = + 2.4%; ES = + 0.1), but it did not reach statistical significance (p = 0.23 for time x group interaction). Fisher’s exact test revealed that the proportion of subjects that experienced reduction in jumping performance was significantly greater in the placebo group than in the creatine group (5 and 1, respectively; p = 0.05) after the intensified training. This was supported by the magnitude-based inference analysis, which demonstrated a *possible negative effect* (50%) in jumping performance in the placebo group, whereas a *very likely trivial effect* (96%) in jumping performance was observed in the creatine group.

## Discussion

Collectively, the present findings suggest that creatine supplementation prevented the progressive training-induced decline in lower-limb performance in professional elite soccer players during pre-season.

The ergogenic effects of creatine supplementation have been shown by several experimental protocols including high-intensity intermittent efforts [2-6]. As soccer shows these characteristics, creatine supplements have often been used by soccer athletes in an attempt to improve their performance. For instance, a survey reported 274 occurrences of creatine-containing supplements during the 2006 *Fédération Internationale de Football Association* (FIFA) World Cup [7]. Possibly, an even higher incidence of creatine users would be found if the survey were extended to the whole season, as this supplement has also been thought to improve the training ability in soccer [36]. Supporting this notion, it was demonstrated that creatine supplementation improved muscle strength in collegiate female soccer players during off-season training [13]. However, the benefits of creatine in soccer remains inconclusive as there are very few data on the effects of chronic supplementation in elite athletes. In this regard, this study shows that chronic creatine supplementation can promote positive effects on lower-limb performance in elite players during a pre-season intensive training, providing applicable evidence that this dietary supplement may benefit professional soccer players.

The main mechanism underlying the beneficial effects of creatine shown in the current study could be a putative increase in the muscle phosphorylcreatine concentration, which could remain elevated during multiple exercise bouts, possibly offsetting the normal decrease in force production that occurs over the course of the training session [5,6,25,37]. In agreement with this speculation, we observed a performance decline in the placebo group, but not in the creatine group, suggesting that creatine supplementation may be effective for maintaining muscular performance during a progressive training program. A similar conclusion was reached by another study, which demonstrated greater improvements in muscular performance following the initial phase of a short-term resistance training overreaching with creatine supplementation in resistance-trained men [37]. Unfortunately, in the present study, we were unable to record the resistance training external load (i.e., external overload in kg and) in order to confirm this suggestion.

This study presents some limitations. First, since our sample was composed of top-level athletes with strict training routines, we were unable to assess muscle creatine content or to perform a battery of physical tests. However, the main goal of this study, which was to test the efficacy of this supplement on lower-limb performance in elite soccer players was effectively achieved. Second, our sample size was relatively small, since the subjects were recruited from a unique club to avoid confounding factors (e.g., different training regimes and diet). To circumvent this issue and prevent potential misinterpretations, different statistical approaches were used, including the magnitude-based inference, which allow detecting any possible changes in the performance that might be relevant in a sports setting. Therefore, a comprehensive analysis of our data based on the individual response, the Fisher’s exact test, the ES, and the magnitude-based inferences, revealed a positive effect of creatine over placebo upon the primary outcome (i.e., jumping performance), despite the lack of an interaction effect detected by the Mixed Model analysis.

## Conclusions

Creatine monohydrate supplementation prevented the decrement in lower-limb muscle power in elite soccer players during pre-season progressive training.

## Abbreviations

CMJ: Countermovement jump; RPE: Perceived exertion; ES: Effect size.

## Competing interests

The authors declare that they have no competing of interest.

## Authors’ contributions

CJG, RH, and GB were significant manuscript writers; MB, AS, ZV, BF, AAC, SJC were significant manuscript revisers/reviewers; CJG, RH, GB, AAC, SJC participated in the concept and design; CJG, MB, AS, ZV, BF were responsible for data acquisition; CJG, RH, GB, AAC, SJC participated in data analysis and interpretation. All authors read and approved the final manuscript.

## References

[B1] Wyss M Kaddurah-Daouk R Creatine and creatinine metabolism Physiol Rev 2000 80 1107 1213 10893433 10.1152/physrev.2000.80.3.1107

[B2] Barber JJ McDermott AY McGaughey KJ Olmstead JD Hagobian TA Effects of combined creatine and sodium bicarbonate supplementation on repeated sprint performance in trained men J Strength Cond Res 2013 27 252 258 23254493 10.1519/JSC.0b013e318252f6b7

[B3] Lee CL Lin JC Cheng CF Effect of caffeine ingestion after creatine supplementation on intermittent high-intensity sprint performance Eur J Appl Physiol 2011 111 1669 1177 21207054 10.1007/s00421-010-1792-0

[B4] Roschel H Gualano B Marquezi M Costa A Lancha AH Jr Creatine supplementation spares muscle glycogen during high intensity intermittent exercise in rats J Int Soc Sports Nutr 2010 7 6 20205834 10.1186/1550-2783-7-6 PMC2825211

[B5] Balsom PD Söderlund K Sjödin B Ekblom B Skeletal muscle metabolism during short duration high-intensity exercise: influence of creatine supplementation Acta Physiol Scand 1995 154 303 310 7572228 10.1111/j.1748-1716.1995.tb09914.x

[B6] Balsom PD Ekblom B Söderlund K Sjödln B Hultman E Creatine supplementation and dynamic high intensity exercise Scand J Med Sci Sports 1993 3 143 149

[B7] Tscholl P Junge A Dvorak J The use of medication and nutritional supplements during FIFA World Cups 2002 and 2006 Br J Sports Med 2008 42 725 730 18308873 10.1136/bjsm.2007.045187 PMC2582332

[B8] Chilibeck PD Magnus C Anderson M Effect of in-season creatine supplementation on body composition and performance in rugby union football players Appl Physiol Nutr Metab 2007 32 1052 1057 18059577 10.1139/H07-072

[B9] Reilly T Training specificity for soccer Int J Appl Sports Sci 2005 17 17 25

[B10] Ostojonic SM Creatine supplementation in young soccer players Int J Sport Nut Exerc Metab 2004 14 95 103 10.1123/ijsnem.14.1.95 15129933

[B11] Mujika I Padilla S Ibañez J Izquierdo M Gorostiaga E Creatine supplementation and sprint performance in soccer players Med Sci Sports Exerc 2000 32 518 525 10694141 10.1097/00005768-200002000-00039

[B12] Cox G Mujika I Tumilty D Burke L Acute creatine supplementation and performance during a field test simulating match play in elite female soccer players Int J Sport Nutr Exerc Metab 2002 12 33 46 11993621 10.1123/ijsnem.12.1.33

[B13] Larson-Meyer DE Hunter GR Trowbridge CA Turk JC Ernest JM Torman SL Harbin PA The effect of creatine supplementation on muscle strength and body composition during off-season training in female soccer players J Strength Cond Res 2000 14 434 442

[B14] Brink MS Visscher C Coutts AJ Lemmink KAPM Changes in perceived stress and recovery in overreached young elite soccer players Scand J Med Sci Sports 2012 22 285 292 21039901 10.1111/j.1600-0838.2010.01237.x

[B15] American College of Sports Medicine American College of Sports Medicine position stand. Progression models in resistance training for healthy adults Med Sci Sports Exerc 2009 41 687 708 19204579 10.1249/MSS.0b013e3181915670

[B16] Markovic G Does plyometric training improve vertical jump height? a meta-analytical review Br J Sports Med 2007 41 349 355 17347316 10.1136/bjsm.2007.035113 PMC2465309

[B17] McGuigan MR Foster C A new approach to monitoring resistance training Strength Cond J 2004 26 42 47

[B18] Impellizzeri FM Rampinini E Coutts AJ Sassi A Marcora SM Use of RPE-based training load in soccer Med Sci Sports Exerc 2004 36 1042 1047 15179175 10.1249/01.mss.0000128199.23901.2f

[B19] Wrigley R Drust B Stratton G Scott M Gregson W Quantification of the typical weekly in-season training load in elite junior soccer players J Sports Sci 2012 30 1573 1580 22852843 10.1080/02640414.2012.709265

[B20] Claudino JG Mezêncio B Soncin R Ferreira JC Couto BP Szmuchrowski LA Pre vertical jump performance to regulate the training volume Int J Sports Med 2012 33 101 107 22187384 10.1055/s-0031-1286293

[B21] Dias JA Dal Pupo J Reis DC Borges L Santos SG Moro AR Borges NG Jr Validity of two methods for estimation of vertical jump height J Strength Cond Res 2011 25 2034 2039 21701288 10.1519/JSC.0b013e3181e73f6e

[B22] Ugrinowitsch C Tricoli V Rodacki AL Batista M Ricard MD Influence of training background on jumping height J Strength Cond Res 2007 21 848 852 17685694 10.1519/R-20162.1

[B23] Lamontagne-Lacasse M Nadon R Goulet EDB Effect of creatine supplementation on jumping performance in elite volleyball players Int J Sports Physiol Perform 2011 6 525 533 21941005 10.1123/ijspp.6.4.525

[B24] Branch JD Effect of creatine supplementation on body composition and performance: a meta-analysis Int J Sport Nutr Exerc Metab 2003 13 198 226 12945830 10.1123/ijsnem.13.2.198

[B25] Izquierdo M Ibañez J González-Badillo JJ Gorostiaga EM Effects of creatine supplementation on muscle power, endurance, and sprint performance Med Sci Sports Exerc 2002 34 332 343 11828245 10.1097/00005768-200202000-00023

[B26] Alves CR Santiago BM Lima FR Otaduy MC Calich AL Tritto AC de Sá Pinto AL Roschel H Leite CC Benatti FB Bonfá E Gualano B Creatine supplementation in fibromyalgia: a randomized, double-blind, placebo-controlled trial Arthritis Care Res 2013 65 1449 1459 10.1002/acr.22020 23554283

[B27] Del Favero S Roschel H Artioli G Ugrinowitsch C Tricoli V Costa A Barroso R Negrelli AL Otaduy MC da Costa LC Lancha-Junior AH Gualano B Creatine but not betaine supplementation increases muscle phosphorylcreatine content and strength performance Amino Acids 2012 42 2299 2305 21744011 10.1007/s00726-011-0972-5

[B28] Gualano B De Salles PV Roschel H Artioli GG Neves M Jr De Sá Pinto AL Da Silva ME Cunha MR Otaduy MC Leite Cda C Ferreira JC Pereira RM Brum PC Bonfá E Lancha AH Jr Creatine in type 2 diabetes: a randomized, double-blind, placebo-controlled trial Med Sci Sports Exerc 2011 43 770 778 20881878 10.1249/MSS.0b013e3181fcee7d

[B29] Hopkins WG A spreadsheet for deriving a confidence interval, mechanistic inference and clinical inference from a p value Sportscience 2007 11 16 20

[B30] Hobson RM Harris RC Martin D Smith P Macklin B Elliott-Sale KJ Sale C Effect of sodium bicarbonate supplementation on 2000-m rowing performance Int J Sports Physiol Perform 2014 9 139 144 23579002 10.1123/ijspp.2013-0086

[B31] Antonio J Ciccone V The effects of pre versus post workout supplementation of creatine monohydrate on body composition and strength J Int Soc Sports Nutr 2013 10 36 23919405 10.1186/1550-2783-10-36 PMC3750511

[B32] Dorling JL Earnest CP Effect of carbohydrate mouth rinsing on multiple sprint performance J Int Soc Sports Nutr 2013 10 41 24066731 10.1186/1550-2783-10-41 PMC3849766

[B33] Hoffman JR Stout JR Williams DR Wells AJ Fragala MS Mangine GT Gonzalez AM Emerson NS McCormack WP Scanlon TC Purpura M Jäger R Efficacy of phosphatidic acid ingestion on lean body mass, muscle thickness and strength gains in resistance-trained men J Int Soc Sports Nutr 2012 9 47 23035701 10.1186/1550-2783-9-47 PMC3506449

[B34] Batterham AM Hopkins WG Making meaningful inferences about magnitudes Sportscience 2005 9 6 13 19114737

[B35] Hopkins WG Probabilities of clinical or practical significance Sportscience 2002 6 sportsci.org/jour/0201/wghprob.htm

[B36] Hespel P Maughan RJ Greenhaff PL Dietary supplements for football J Sports Sci 2006 24 749 761 16766503 10.1080/02640410500482974

[B37] Volek JS Ratamess NA Rubin MR Gómez AL French DN McGuigan MM Scheett TP Sharman MJ Häkkinen K Kraemer WJ The effects of creatine supplementation on muscular performance and body composition responses to short-term resistance training overreaching Eur J Appl Physiol 2004 91 628 637 14685870 10.1007/s00421-003-1031-z

